# Implications of Storing Urinary DNA from Different Populations for Molecular Analyses

**DOI:** 10.1371/journal.pone.0006985

**Published:** 2009-09-10

**Authors:** Angela Cannas, Glendah Kalunga, Clare Green, Ludovica Calvo, Patrick Katemangwe, Klaus Reither, Mark D. Perkins, Leonard Maboko, Michael Hoelscher, Elizabeth A. Talbot, Peter Mwaba, Alimuddin I. Zumla, Enrico Girardi, Jim F. Huggett, for the TB trDNA consortium

**Affiliations:** 1 National Institute for Infectious Diseases L. Spallanzani, IRCCS, Roma, Italy; 2 UNZA-UCLMS Research & Training Project, University Teaching Hospital, Lusaka, Zambia; 3 Centre for Infectious Diseases and International Health, Windeyer Institute for Medical Sciences, University College London, London, United Kingdom; 4 NIMR-Mbeya Medical Research Programme, Mbeya, Tanzania; 5 Foundation for Innovative New Diagnostics (FIND), Geneva, Switzerland; 6 Department of Infectious Diseases and Tropical Medicine, Klinikum of the University of Ludwig-Maximilians-Munich, Munich, Germany; Université Pierre et Marie Curie, France

## Abstract

**Background:**

Molecular diagnosis using urine is established for many sexually transmitted diseases and is increasingly used to diagnose tumours and other infectious diseases. Storage of urine prior to analysis, whether due to home collection or bio-banking, is increasingly advocated yet no best practice has emerged. Here, we examined the stability of DNA in stored urine in two populations over 28 days.

**Methodology:**

Urine from 40 (20 male) healthy volunteers from two populations, Italy and Zambia, was stored at four different temperatures (RT, 4°C, −20°C & −80°C) with and without EDTA preservative solution. Urines were extracted at days 0, 1, 3, 7 and 28 after storage. Human DNA content was measured using multi-copy (ALU J) and single copy (TLR2) targets by quantitative real-time PCR. Zambian and Italian samples contained comparable DNA quantity at time zero. Generally, two trends were observed during storage; no degradation, or rapid degradation from days 0 to 7 followed by little further degradation to 28 days. The biphasic degradation was always observed in Zambia regardless of storage conditions, but only twice in Italy.

**Conclusion:**

Site-specific differences in urine composition significantly affect the stability of DNA during storage. Assessing the quality of stored urine for molecular analysis, by using the type of strategy described here, is paramount before these samples are used for molecular prognostic monitoring, genetic analyses and disease diagnosis.

## Introduction

Molecular diagnosis, where nucleic acids are measured in the context of a disease, is a multistep process requiring collection, storage, and extraction of a sample prior to analysis. Diagnostic samples come in many guises ranging from solid biopsies to liquid material and their sampling can require highly invasive procedures. Urine represents the ideal clinical sample, being easy to collect and homogenous, so less likely to suffer from sample bias that may affect other material, such as a biopsy. The existence of DNA in excreted urine is well established and this represents a potentially useful source of genetic material. DNA arising from cells shed into the lumen of genitourinary tract can be used for the detection of genetic anomalies and neoplasia associated with the bladder, prostate or kidney [1], [2]. In addition, some infections are accompanied by the appearance in the urine of the causative virus or bacterium where the kidney or bladder are involved in the pathogenesis, or as a consequence of loss of the renal barrier integrity [3].

The molecular detection from urine of pathogens that infect the genitourinary system is frequently performed [4]. The use of urine as a sample for non-genitourinary infections has also been successfully reported in tuberculosis [5], leishmaniasis [6] and malaria [7], although in this context it is not widespread. One of the reasons for this may be due to the high variability in the reported efficacy of detection. Tuberculosis (TB) is a case in point; of seven studies reporting amplification of *Mycobacterium tuberculosis* DNA using urine as a diagnostic sample [5], [8], [9], [10], [11], [12], [13], detection sensitivities range from <30% [9], [10] to >70% [5], [8] (reviewed in [14]). Certainly, one of the variables potentially contributing to this variation was urine storage.

Reports on how best to store urine for molecular detection are sparse. Studies whose primary outcome was the measurement of bacterial DNA have concluded that storage at 4°C for up to 30 days with EDTA [15], or for 1 week without EDTA [16] did not affect molecular detection. Studies whose primary outcome was the recovery and analysis of human DNA have concluded that storing urine at room temperature with sodium azide over 30 days [17] or at −20°C with EDTA over 72 days [18], provides the best storage method. Furthermore, in other studies, useful human molecular analyses have been obtained from urine stored without preservative at −20°C for up to 25 years [19] and up to 7 years [20]. Whilst recorded differences in optimal storage conditions are also a function of the extraction method, outcome measure and the nature of the nucleic acid measured (for example, cell-free or cellular, single or double stranded, RNA or DNA), the recommendations produced by these studies vary, suggesting differences in the stability of their respective urine samples.

To investigate this in detail, we measured the stability of urinary DNA over 28 days, at four commonly examined storage temperatures, with and without the urine preservative EDTA at two geographically distinct sites. Our findings reflect the contradictions in the existing literature, as we observed significant variability in urinary DNA stability irrespective of storage conditions when sampled from different sources. We would recommend that researchers undertaking molecular analysis of urine use this type of approach to either conduct their own stability study or assess the status of their banked samples. These findings apply to all cases (for example, molecular diagnosis, prognostic monitoring, genetic/epidemiological screening and forensic testing) where molecular analysis of stored urine provides the outcome measure.

## Materials and Methods

### Ethics Statement

All study participants gave written informed consent in accordance with local guidelines and the study was approved by The University of Zambia Research Ethics Committee in Zambia and the Ethics Board of the National Institute for Infectious Diseases Lazzaro Spallanzani in Italy.

### Study participants

20 (10 male) healthy volunteers were enrolled at the National Institute for Infectious Diseases L. Spallanzani (INMI), Rome, Italy and an additional 20 (10 male) healthy volunteers were enrolled at University Teaching Hospital (UTH), Lusaka, Zambia. A second group of 20 (10 male) healthy volunteers were enrolled at a later date at INMI to further investigate the findings when urine is stored at −20°C. Participants were confirmed to be free from urinary tract infection using a commercial dipstick (Multistix, Bayer, Newbury, UK: UTH), or automated (Aution Max, Menarini, Italy & Sysmex UF100, Dasit, Italy: INMI).

### Urine specimen collection and storage

Urine specimens were collected between 0700 and 1000 hours and stored as per storage schedule (described below). Urine processing and sampling was performed within 20 minutes of collection. 50 ml of mid-stream urine was collected from each volunteer, and immediately separated into two 25 ml storage fractions. One fraction was stored as undiluted urine and the other with ethylenediaminetetracetic-Tris-HCl, pH 8.5 (EDTA solution). In a small pilot investigation in Italy, we determined that the recovery of human DNA from samples stored with 40 mM or 10 mM final concentration of EDTA at −20°C for 7 days showed the same considerable degradation (data not shown). This was contrary to the data produced from a previous study by Milde *et al*. which investigated 40 mM EDTA for storage of urine over three months [18]. In order to examine the effect of EDTA on urinary DNA stability over time under a number of conditions, and to replicate the Milde study, EDTA was added to a final concentration of 40 mM in the Italian arm of the study. In the Zambian arm of the study EDTA at 10 mM was used as this corresponds to the concentration of EDTA commonly present in commercially available urine preservative kits [21], [22], [23]. These are used for sample transport and are potentially good candidates for the increasingly popular biobanking of samples. To avoid the potential of experimentally induced trends, and for logistical reasons, sampling was staggered so that the full time course for 20 volunteers took approximately three months; consequently extractions of different volunteer urine samples from different storage times were frequently performed simultaneously. Furthermore all the same time points under different storage conditions for the same individual's urine sample were extracted together. The complexities of the study and logistical reasons prevented us from examining both preservative concentrations at both sites during the allotted timeframe. One ml aliquots of both storage fractions were stored at room temperature (∼18–22°C in Italy, ∼19–25°C in Zambia), +4°C, −20°C, and −80°C. The stored urine aliquots were thawed and processed on days 1, 3, 7 and 28 following collection. An additional aliquot of urine was immediately processed for DNA extraction (day 0). To further investigate the specific findings in the Italian samples at −20°C, we repeated the experiment using a further 20 volunteers and investigated storage without additive and with 40 mM EDTA over seven days.

### DNA isolation

DNA was isolated from urine following a protocol to specifically purify small nucleic acids previously developed at University College London in collaboration with Xenomics Inc. (New York, USA). DNA was captured from 1 ml of urine by adding 30 µl of Q-sepharose (GE-Healthcare^™^, Little Chalfont, UK) and incubated at room temperature for 30 minutes with constant mixing. The Q-sepharose was pelleted and washed with 2×1 ml 400 mM NaCl (Sigma, Dorset, UK) followed by 1×1 ml 550 mM NaCl. Captured DNA was eluted from the sepharose using 560 µl of lysis buffer (AVL) and further purified using the QiAmp Viral Mini kit following manufacturer's instructions (Qiagen, Valencia, USA). DNA was eluted into 100 µl of PCR grade water and stored in 25 µl aliquots at −80°C before PCR analysis.

### Real time PCR

Three quantitative real-time PCR reactions were performed during this study following the *M*inimum *I*nformation for publication of *Q*uantitative real-time PCR *E*xperiments (MIQE) guidelines [24], and further information on data analysis and methodology is summarized in the supplementary tables S1 [Supplementary-material pone.0006985.s002] [Supplementary-material pone.0006985.s003] to S4. To ensure that different sites conducted comparable analysis, all reactions and plasmid standards were developed at University College London and quality assessed before shipping to the respective experimental sites. Repeat quality assessment was then performed at each experimental site to ensure stable transport prior to qPCR analysis. Consequently all experimentation used exactly the same standards, primer and probe batches. Amplification of the single copy gene for human toll-like receptor 2 (TLR2) (750 nM forward primer TTGCTGGACTTACCTTCCTTG, 750 nM reverse primer TGACTTCAAACTTTTTGGCTCA) and the multi copy human target ALU J (600 nM forward CAACATAGTGAAACCCCGTCTCT & 600 nM reverse primer GCCTCAGCCTCCCGAGTAG) were targeted to measure human DNA content, and the SPUD inhibition assay to assess inhibition. The SPUD reaction, which has been previously described [25], was performed on all extracts prior to other qPCR analyses. All real-time qPCR reactions were conducted in 12.5 µl volumes in a Rotorgene 6000 thermocycler with amplification measured by excitation at 470 nm and acquisition of fluorescence at 510 nm following each extension. PCR efficiencies were estimated using 10 fold dilution series (comprising linearised plasmid containing amplicon) according to the formula E = 10^(−1/slope)−1^. Evaluation of the presence of inhibitors in the DNA extracts was performed using the SPUD protocol as previously described [26]. Inhibition assessment was performed on 5 µl isolated DNA and 0.5 µl of isolated DNA (equivalent 50 µl and 5 µl volumes of urine respectively). Inhibition reactions were assessed using 1,000 copies of the SPUD amplicon. Following inhibition assessment ALU J and TLR2 reactions were performed using 0.5 µl of DNA extract (equivalent to 5 µl of urine).

### Pico green measurement of DNA extracts

PicoGreen assessments were performed following manufacturers' instructions (Invitrogen, Carlsbad, CA, USA). A standard curve (150, 100, 75, 50, 25, 10 & 5 pg/µl) was generated using lambda DNA included by the manufacturer. 20 µl analysis volumes comprising 10 ul of lambda standard or undiluted samples, and 10 µl Quant-iT PicoGreen reagent (diluted 200 fold using 1X TE) were analysed. Fluorometric analysis using the rotorgene 6000 was performed using the following parameters: a 2 minute incubation at 50°C followed by ten ten second incubations at 60°C and fluorescence measured by excitation at 470 nm and acquisition at 510 nm following each 60°C incubation.

### Data presentation and statistical analysis

All data was assessed for normality using the D'Agostino & Pearson omnibus normality test. Day 0 data was compared using MannWhitney U test. To assess the effect of storage on DNA stability, qPCR and pico green data was log transformed and 95% confidence intervals (CI) calculated. Exponentiated data was plotted as geometric mean +/−95% CI. ALU J and TLR2 data was compared with pico green analysis using the Spearman rank test.

## Results

### Participant's age and urine analysis

At INMI the mean age of participants was 37.3 years (range 26–60 years) for the first group and 38.1 (range 28–55 years) for the second group. At UTH the mean age was 24.8 years (range 7–45 years). All volunteers had urinary protein, nitrite, glucose, ketone, pH, specific gravity, urobilirubin, and haemoglobin within normal reference ranges. Bacteria, blood cells (erythrocytes, leucocytes) and epithelial cells were also within the normal reference ranges for all donors, except three menstruating females whose urine contained traces of blood.

### PCR inhibition

PCR inhibition was detected in all samples when 50 µl effective volume of urine (5 µl of extract) was used. A ten-fold dilution of the extracted DNA samples removed this inhibition from all extracts and the remainder of the analysis was performed using the effective urine volume of 5 µl per reaction.

### DNA measurements from fresh human urine

**Figure 1:**
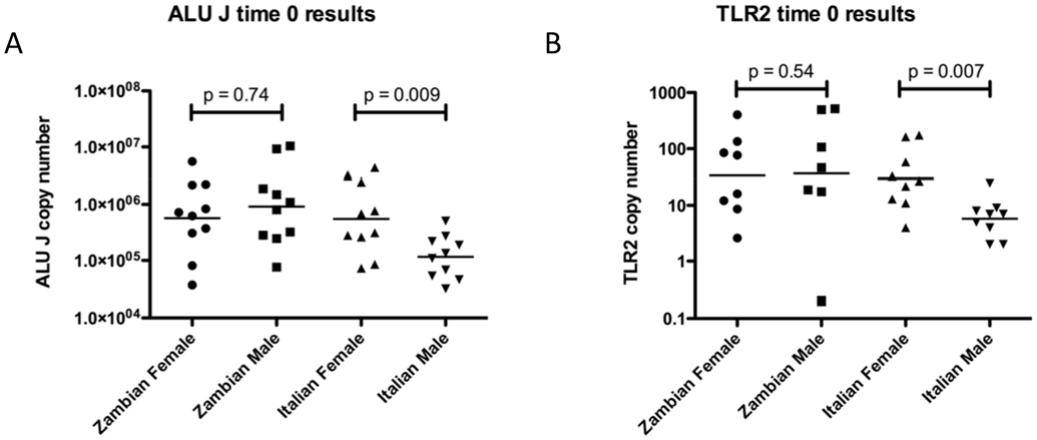
Baseline (day 0) assessment of human DNA. The amplification of multicopy ALU J sequence (A) and single copy TLR2 (B) sequences were comparable across the populations. Italian males had significantly less human DNA at baseline than females. No such sex difference was observed for the Zambian urines. Scatter plot of baseline data showing geometric mean.

**Figure 2:**
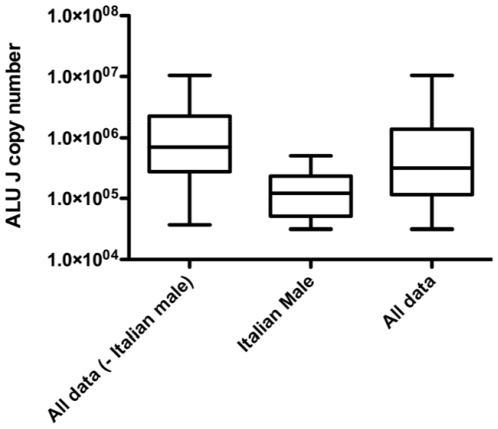
Baseline (day 0) ALU J DNA sequences are log normally distributed. Italian male data contains less human DNA compared to females but still falls within the range of the all other baseline data. The data remain log normally distributed when both sites and sexes are combined. Box and whisker plots showing median, 25^th^ and 75^th^ percentiles and range.

The base line DNA measurement using the ALU J assays ranged from 30,000 to 10,450,000 copies/ 5 µl of urine. All data gave a log normal distribution. Furthermore the geometric mean was 430,000 copies less (p = 0.009) in males from Italy (mean 34.5 years) than in Italian females (mean 40.1 years) (figure 1A). The TLR2 data measurement demonstrated a similar result to that of ALU J (figure 1B), but with considerably lower copies as expected for a single copy target. There was no difference between male (mean 26.5 years) and female (mean 23 years) DNA quantity from Zambian participants or between Italian females and the all Zambian data (figure 1A and 1B). Although the Italian male data was different it fell within the range of the other baseline data (figure 2). Furthermore the data remained log normally distributed when both sites and sexes were combined.

### Effects of storage temperature on stability of DNA in urine

**Figure 3:**
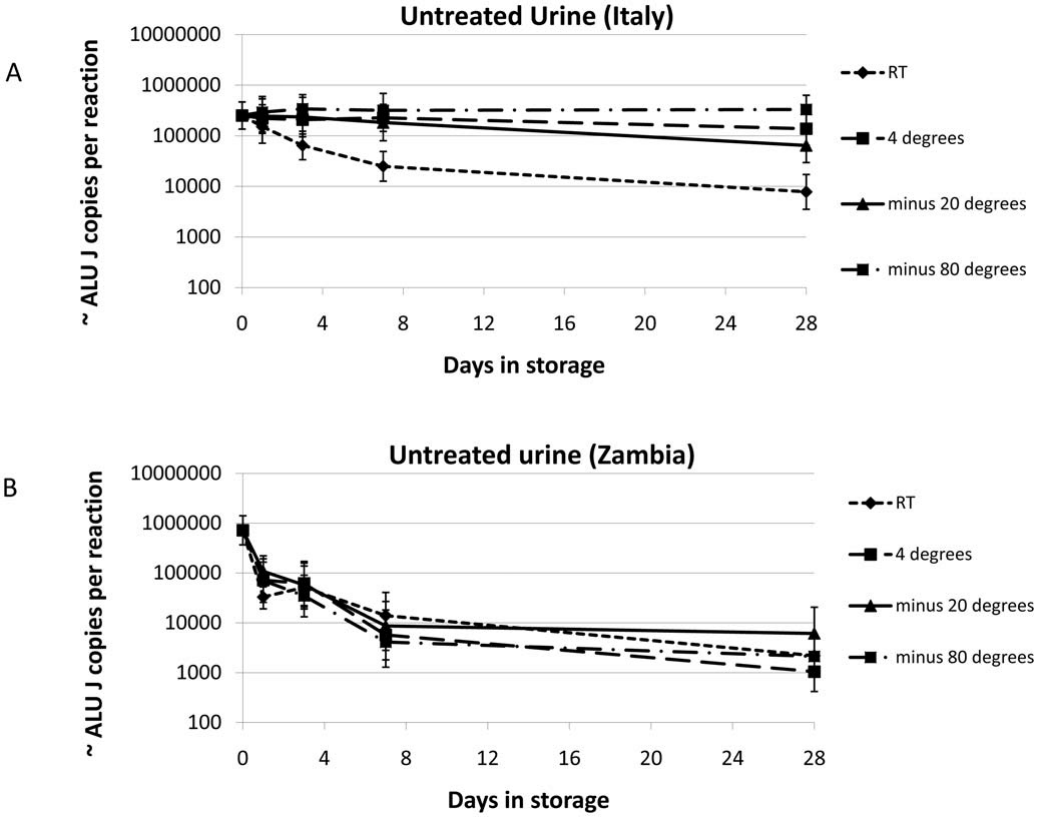
The effect of storage temperature on untreated urine from two populations over 28 days. Human DNA, as measured by the ALU J assay, is quite stable in all temperatures in Italy (A) except room temperature where considerable degradation is observed. In Zambia (B) human DNA is rapidly lost at all of the temperatures examined. Geometric means +/−95% confidence intervals are plotted for each treatment.

When the untreated urine from Italian samples was stored at room temperature on average ∼96.9% of the original ALU J signal was lost by day 28 (figure 3A). On average, ∼74.6% was lost after 28 days when stored at −20°C, ∼45.3% at 4°C and the degradation was completely halted when stored at −80°C. In contrast, the loss of ALU J signal in the Zambian urines without preservative was on average >99% after 28 days with all storage temperatures (figure 3B). Even after only 7 days in storage the loss was on average ∼98.8%. The TLR2 data was comparable to the ALU J data, although because the starting copy was much lower, any decrease in stability generally led to complete loss of signal (data not shown). There was no difference in the degradation trends between samples from the male and female volunteers at either site.

### Effects of EDTA and storage temperature on stability of DNA in urine

**Figure 4:**
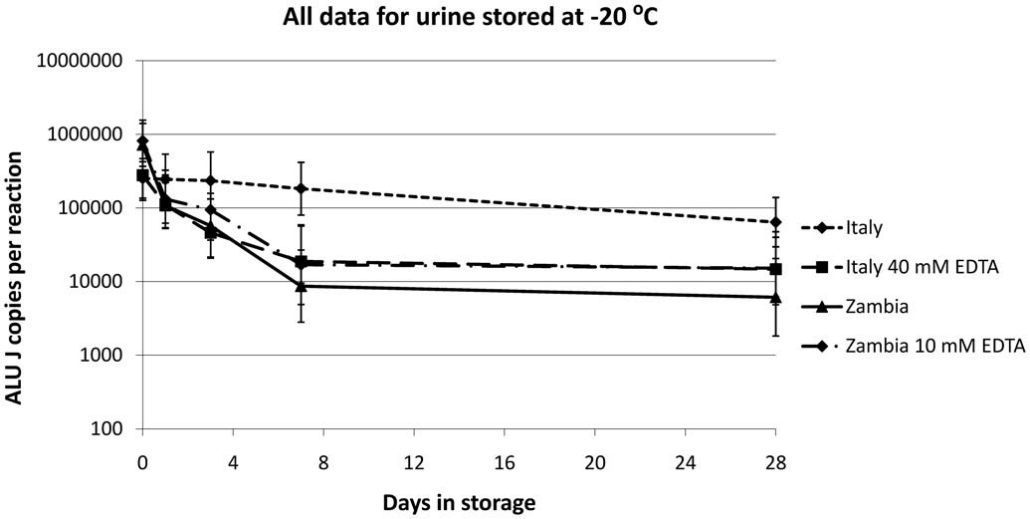
Direct comparison of human DNA stability in samples stored at −20°C over 28 days. The Italian urines stored with 40 mM EDTA at −20 C show the same biphasic degradation of human DNA observed in all Zambian urines. This biphasic degradation of the Zambian urines occurs in both the presence and absence of EDTA. Geometric means +/−95% confidence intervals are plotted for each treatment.

The addition of 40 mM EDTA to the Italian samples prevented degradation for 28 days when the samples were stored at room temperature, 4°C and −80°C; with an average loss of only 1.6% compared to baseline (data not shown). However, for urines stored at −20°C the addition of 40 mM EDTA did not prevent a ∼94.7% loss of DNA by day 28 (figure 4). With the Zambian samples, the addition of 10 mM EDTA solution, reflecting many commercially available urine preservation kits [21], [22], [23], had no stabilising effect regardless of storage temperature (data not shown). At this site, an average of ∼98.9% of the baseline DNA quantity was lost by day 7, increasing to ∼99.3% by day 28.

### Detailed assessment of storage at −20°C

When the samples were stored at −20°C the untreated Italian sample showed a linear degradation of ∼74.6% of the original amount over 28 days (figure 4). This was different from the biphasic degradation observed when the Italian samples were stored with 40 mM EDTA solution or in all storage conditions with the Zambian samples. The degradation seen in the Italian samples with EDTA resembled data from a preliminary study we performed at the same site comparing the effect of 10 mM and 40 mM EDTA on urine samples stored at −20°C for 7 days (data not shown). Neither 10 mM or 40 mM EDTA concentration stabilised urinary DNA. In our current study, the biphasic degradation observed was characterised by a more rapid loss of the ALU J signal to day seven, followed by a cessation or much reduced degradation to 28 days (figure 4). The Zambian urines showed the same trend in degradation at −20°C regardless of the presence of EDTA (figure 4).

**Figure 5:**
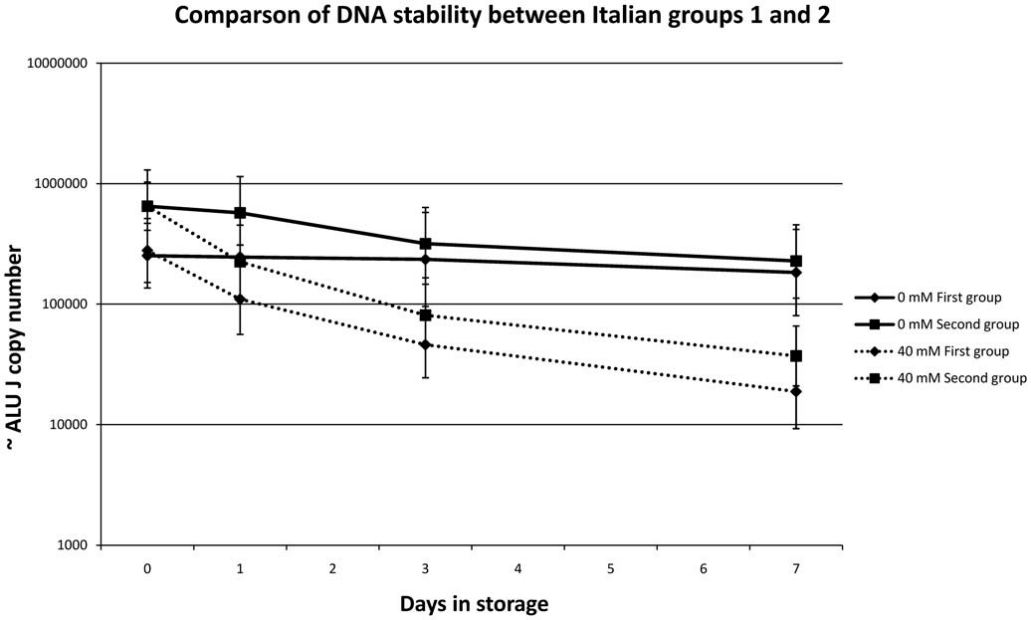
Comparison of DNA stability between Italian groups 1 and 2. Stability of human DNA in urine over 7 days compared between groups 1 and 2 demonstrate that the stability trend observed for the second analyses is highly consistent with the first. Specifically the degradation when urine is stored with EDTA is almost identical between the two groups. Geometric means +/−95% confidence intervals are plotted for each treatment.

**Figure 6:**
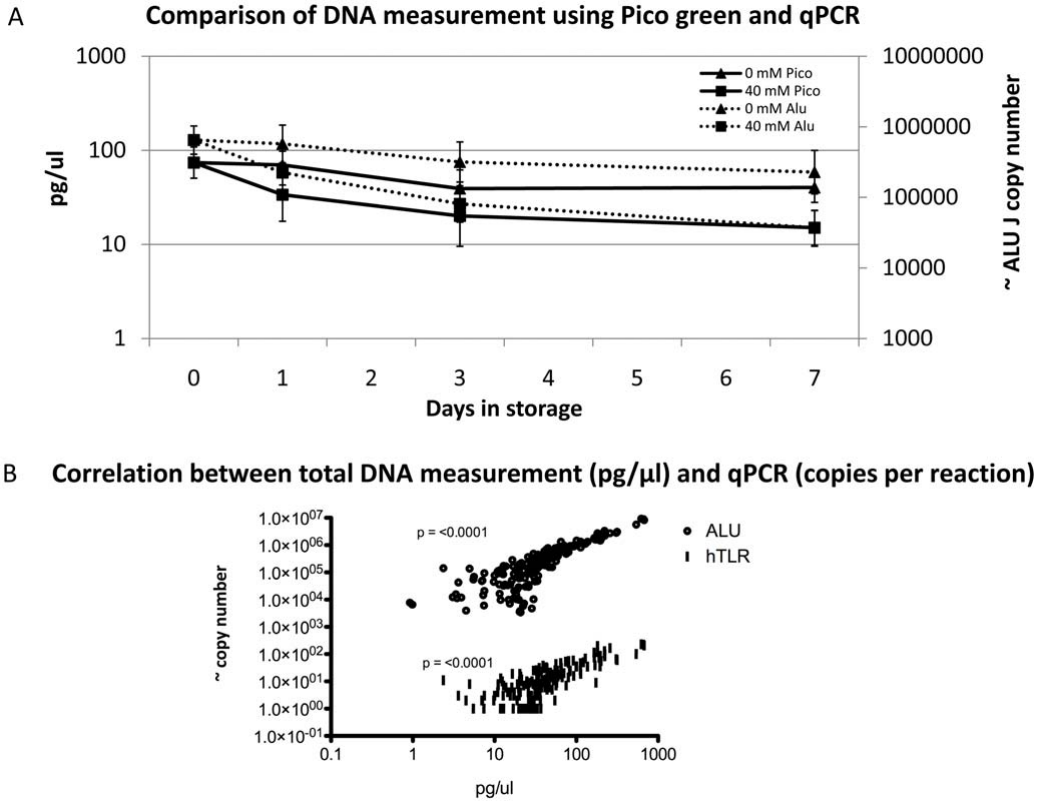
Comparison of DNA measurement using Pico green and qPCR. The stability of human and total DNA in urine stored at −20 C with 40 mM EDTA and without EDTA shown the same trends (A). Geometric means +/−95% confidence intervals are plotted for each treatment. Measurement with both human qPCR targets correlate with the direct measurement of total nucleic acids using pico-green (B). QPCR using these targets is an appropriate surrogate measure for total DNA and has a greater dynamic range than pico-green for which the lower threshold for accurate measurements is approximately 10 pg/µl.

To confirm this finding the Italian analysis was repeated on a second group of volunteers by storing 20 healthy urine samples for seven days and assessed using the ALU J and TLR2 qPCR assays as well as by measuring total DNA using pico green. When urine was stored at −20 with 40 mM EDTA the degradation in the second group was identical to the first group (figure 5). Furthermore assessment of these samples using the Pico Green DNA measurement method confirmed the qPCR findings at −20°C with 40 mM EDTA (figure 6A) and supported the use of qPCR human targets as a surrogate measurement of total urinary DNA (figure 6B). We found that ∼100,000 copies of ALU J and ∼100 copies of TLR2 are equivalent to ∼47 pg and ∼65 pg of total DNA respectively.

## Discussion

Our data show that the stability of human DNA in urine is dependent on geographic origin. The variation in stability of human DNA is presumably due to differences in the urinary matrix between locations. Of the several factors we examined (and potentially important in determining the stability of human DNA in urine): sex, two geographically distinct sites, addition of EDTA as a preserving solution, storage temperature and duration; only study location and the addition of EDTA correlated with stability.

Baseline DNA measurements showed that Italian, but not Zambian, males had decreased urinary DNA compared with females, although this remained within the log normal distribution. A similar reduction in the amount of human urinary DNA at baseline in males has been recorded for studies in USA [17], [27], Germany [18], [20], but was not observed in healthy individuals from Russia [28] or the Zambian individuals in this study.

Irrespective of sex, the Italian and Zambian samples had comparable distribution at baseline, which ranges over two orders of magnitude. A considerable variation in total nucleic acid content of urine has been previously documented, for example, 27–189 ng/ml [28], 50–200 ng/ml [18]. By using qPCR for multi- and single target human genes as a surrogate measure for urinary DNA content we have accurately quantified this range in a number of healthy individuals. The ALU J assay amplifies a family of short interspersed nuclear elements from the ALU group which comprise over 10% of the human genome [29]. Therefore, ALU J provides a useful approximate measure of genomic DNA which is able to record differences in quantity of two orders of magnitude, and due to the high initial copy number, has the capacity to record considerable degradation of human DNA over time. Furthermore, the similarities between the distribution of our two sites suggests this approach provides a facile tool to measure urinary DNA content applicable to ongoing studies and bio-banks from different populations.

The addition of EDTA to a final concentration of 10 mM did not stabilise urinary DNA in Zambia. Zambian urines essentially demonstrated a biphasic >2 log degradation over time, irrelevant of storage temperature or addition of EDTA. The urine preservative EDTA is commonly employed at a final concentration of 10 mM by commercially available urine transport tubes sold specifically for down-stream molecular analysis [15], [21], [23]. Although this approach is highly suitable to large scale studies in less developed countries, we would not recommend this concentration of EDTA to be used in further studies at our Zambian site. At our Italian site, the addition of EDTA did improve urinary DNA stability under most, but not all, conditions examined. EDTA added to a final concentration of 40 mM did not stabilise the Italian urines stored at −20°C for 28 days, and actually decreased stability compared to samples containing no preservative. These findings were contrary to the previous report [18] on which this aspect of the study was modelled. In this study, we have not investigated whether 40 mM EDTA would have stabilised Zambian urinary DNA, however, we do clearly demonstrate that Zambian urinary DNA is far less stable under storage than Italian urinary DNA. Furthermore, it is clear that urine DNA stability observed in one population cannot be assumed to be representative of another.

An additional question posed by this study is what is the source of this degradation? At this stage it is unclear, whether the reason for this is due to genetic, dietary, climatic or other differences, observed in the Zambian samples. Our most informative result may be that the Italian urines are stored at −20°C degrade in the presence of EDTA. This degradation is comparable to that observed in all the Zambian samples (figure 4) and is unique to this temperature under these conditions: addition of EDTA completely stabilises Italian urinary DNA both at temperatures above and below −20°C. The storage temperature, addition of EDTA and raised pH suggests that the observed degradation is not due to nuclease digestion, but rather an alternative mechanism like the eutectic phenomenon. This is certainly able to synthesis nucleic acids, and increasing solute concentration has also been reported to degrade them [30] and may explain this unexpected observation. Further work is required to test this theory and establish if this or other mechanisms are responsible for the other nucleic acid degradation observed in this study.

Whatever the cause of the observed differences in degradation it is clear that the storage methods investigated, and previously used [16], [17], [18], are not universally suitable. This is likely to explain the contradictory conclusions of previous stability studies and may, in part, contribute to differences observed in the TB diagnostic studies using urine as a clinical sample, described above. A method for universal storage of urine optimised for subsequent nucleic acid analysis remains to be described and our findings have implications for bio-bank setup and storage, which are currently gaining prominence. For example the UK bio-bank stores urine for a wide range of measurements including DNA analysis; urine is stored at −80°C and in liquid N_2_ [31], [32]. If the UK sample stability reflects our Italian volunteers, or the Dutch female urines stored for 15–25 years before molecular analysis [19], then the −80°C storage will be suitable for subsequent DNA analysis. If they resemble the Zambian samples, then it is likely that the DNA suitability for molecular analyses will be greatly reduced even after one week in storage.

In summary, we have demonstrated that urinary DNA stability can be highly variable. Further work is required to identify the source of this variability and the case of the degradation. However our findings likely explain why there has been considerable disagreement in the literature as to how best to store urine for molecular analysis. We also present a novel solution, using the ALU J assay, to assess the status of existing urine bio-banks for DNA degradation and the suitability of the chosen method of storage. These findings and methodologies should be considered for collection, shipping and storage of urine for subsequent molecular diagnosis, therapy monitoring and genetic analysis.

## Supporting Information

Table S1 MIQE checklist. MIQE checklist for quantitative PCR assays used in this study for reviewers reference.
(0.03 MB XLS)

Table S2 qPCR oligonucleotides and hardware
(0.03 MB DOC)

Table S3 Reaction conditions and thermocycling
(0.03 MB DOC)

Table S4 qPCR validation and data analysis
(0.03 MB DOC)
