# Supplementary material for: Implications of Storing Urinary DNA from Different Populations for Molecular Analyses
Source: PLoS One. 2009 Sep 10;4(9):e6985. doi: 10.1371/journal.pone.0006985 (PMC2735781; doi:10.1371/journal.pone.0006985)
Supplement: Table S2 — qPCR oligonucleotides and hardware (0.03 MB DOC) [file pone.0006985.s002.doc]

Supplementary table 2: qPCR oligonucleotides & hardware

| Assay | Manufactured by | Purification method | PCR tubes | Reaction set up | qPCR machine & software |
| --- | --- | --- | --- | --- | --- |
| All assays | Sigma Genosys | DST | 0.2 ml qPCR, strips of 4 (Corbett Life Sciences, #3001-002) | Manual | Rotorgene 6000 & series software v1.7 (Corbett Life Sciences) |
