# Supplementary material for: Implications of Storing Urinary DNA from Different Populations for Molecular Analyses
Source: PLoS One. 2009 Sep 10;4(9):e6985. doi: 10.1371/journal.pone.0006985 (PMC2735781; doi:10.1371/journal.pone.0006985)
Supplement: Table S3 — Reaction conditions and thermocycling (0.03 MB DOC) [file pone.0006985.s003.doc]

Supplementary table 3: Reaction conditions and thermocycling

| Assay | PCR mastermix | Reaction parameters | | | | |
| --- | --- | --- | --- | --- | --- | --- |
|  |  | Annealing temperature | 95 ˚C | Annealing | 72 ˚C | Wavelength excite/acquire |
| TLR2 | 750 nM F&R primers, 1 x SYBR green MM (Qiagen), 250 ng/µl tRNA (Sigma) | 57 ˚C | 10 sec | 20 sec | 30 sec | 470 nm/ 510 nm |
| ALU J | 600 nM F&R primers, 1 x SYBR green MM (Qiagen), 250 ng/µl tRNA (Sigma) | 58 ˚C | 10 sec | 20 sec | 20 sec | 470 nm/ 510 nm |
