# Supplementary material for: Implications of Storing Urinary DNA from Different Populations for Molecular Analyses
Source: PLoS One. 2009 Sep 10;4(9):e6985. doi: 10.1371/journal.pone.0006985 (PMC2735781; doi:10.1371/journal.pone.0006985)
Supplement: Table S4 — qPCR validation and data analysis (0.03 MB DOC) [file pone.0006985.s004.doc]

Supplementary table 4: qPCR validation and data analysis

| Assay | Study site | Average PCR efficiency (n) | 95 % CI PCR efficiency | r^2^ | Linear dynamic range (% CV) | Cq of the NTC | 95 % CI for Cq at min |
| --- | --- | --- | --- | --- | --- | --- | --- |
| TLR2 | Zambia | 95.6 (11) | ± 0.056 | 0.9993 | 50,000 (10.3) to 5 (64.3) | NA | NA |
|  | Italy | 97.8 (23) | ± 0.054 | 0.9975 | 50,000 (12.6) to 5 (54.7) | NA | NA |
| ALU J | Zambia | 97.3 (19) | ± 0.033 | 0.9967 | 5,000,000 (13.2) to 500 (17.3) | 29.09 | ± 0.03 |
|  | Italy | 95.8 (21) | ± 0.035 | 0.9975 | 5,000,000 (14.8) to 500 (15.5) | 30.97 | ± 0.33 |

n = number of runs. Standards were included as duplicates in each run.

NA = no amplification.
